# A closed-body preclinical model to investigate blast-induced spinal cord injury

**DOI:** 10.3389/fnmol.2023.1199732

**Published:** 2023-06-13

**Authors:** Carly Norris, Justin Weatherbee, Susan Murphy, Izabele Marquetti, Lana Maniakhina, Alan Boruch, Pamela VandeVord

**Affiliations:** ^1^School of Biomedical Engineering and Sciences, Virginia Tech, Blacksburg, VA, United States; ^2^Department of Biomedical Engineering and Mechanics, Virginia Tech, Blacksburg, VA, United States; ^3^Veterans Affairs Medical Center, Salem, VA, United States; ^4^Edward Via College of Osteopathic Medicine, Carolinas Campus, Spartanburg, SC, United States

**Keywords:** glial activation, neuroinflammation, closed-body model, spinal cord injury, blast

## Abstract

Blast-induced spinal cord injuries (bSCI) are common and account for 75% of all combat-related spinal trauma. It remains unclear how the rapid change in pressure contributes to pathological outcomes resulting from these complex injuries. Further research is necessary to aid in specialized treatments for those affected. The purpose of this study was to develop a preclinical injury model to investigate the behavior and pathophysiology of blast exposure to the spine, which will bring further insight into outcomes and treatment decisions for complex spinal cord injuries (SCI). An Advanced Blast Simulator was used to study how blast exposure affects the spinal cord in a non-invasive manner. A custom fixture was designed to support the animal in a position that protects the vital organs while exposing the thoracolumbar region of the spine to the blast wave. The Tarlov Scale and Open Field Test (OFT) were used to detect changes in locomotion or anxiety, respectively, 72 h following bSCI. Spinal cords were then harvested and histological staining was performed to investigate markers of traumatic axonal injury (β-APP, NF-L) and neuroinflammation (GFAP, Iba1, S100β). Analysis of the blast dynamics demonstrated that this closed-body model for bSCI was found to be highly repeatable, administering consistent pressure pulses following a Friedlander waveform. There were no significant changes in acute behavior; however, expression of β-APP, Iba1, and GFAP significantly increased in the spinal cord following blast exposure (*p* < 0.05). Additional measures of cell count and area of positive signal provided evidence of increased inflammation and gliosis in the spinal cord at 72 h after blast injury. These findings indicated that pathophysiological responses from the blast alone are detectable, likely contributing to the combined effects. This novel injury model also demonstrated applications as a closed-body SCI model for neuroinflammation enhancing relevance of the preclinical model. Further investigation is necessary to assess the longitudinal pathological outcomes, combined effects from complex injuries, and minimally invasive treatment approaches.

## 1. Introduction

Explosions are the leading cause of all combat-related injuries and account for approximately 75% of all combat-related spinal trauma, resulting in higher injury severity scores and longer hospital stays (Owens et al., 2008; Blair et al., 2012; Schoenfeld et al., 2013; Alizadeh et al., 2019). As the use of improvised explosive devices (IEDs) on the battlefield continues to increase, there is a great need for further investigation of blast-induced spinal cord injuries (bSCI) (Wolf et al., 2009). The mechanism of injury can be broken down into four categories: (1) primary injury resulting from the blast wave alone, (2) secondary injury from displaced fragments during the explosion, (3) tertiary injury from collision of the body with a surface or large object, and (4) quaternary injury from burns, gasses, or asphyxiation (Wolf et al., 2009; Kang et al., 2012). A majority of military bSCI cases are complex, resulting from a combination of the above mechanisms with the most prevalent injuries in either the lumbar and thoracic regions, and almost one-third of soldiers with bSCI sustained injuries in both the thoracic and lumbar (thoracolumbar) regions (Schoenfeld et al., 2013; Bernstock et al., 2015). Patient care typically focuses on the treatment of the fracture; however, we hypothesize that similar to blast-induced neurotrauma, the blast wave causes a unique pathological response in the spinal cord that likely contributes to these complex injuries and warrants a more specialized treatment for bSCI patients.

This work investigated the unique pathological response of the spinal cord following blast exposure using a novel preclinical model for bSCI. There are many preclinical SCI models, including: contusion, transection, compression, distraction/dislocation, existent, and aspiration subtypes where procedures typically include laminectomy prior to testing (Cheriyan et al., 2014; Sharif-Alhoseini et al., 2017). Laminectomy is preferred in these cases to have unobstructed access to the spinal cord and achieve desired repeatability. However, a closed-body injury without laminectomy more appropriately translates the mechanism of spinal cord injury due to blast exposure and mitigates any confounding physiological effects from surgical procedures. Therefore, there is a need to recognize translational closed-body spinal cord injury models as a new subset, which is essential for studying the response following blast exposure.

A closed-body SCI model was developed by del Mar et al. to investigate the progression of post-traumatic axonal pathologies in mice following exposure to directed, high-pressure air blasts at 20 and 70 psi for 15 to 30 ms durations, respectively. Microtubule breakage, calpain activation, neurofilament compaction, and progressive motor deficits were detected (del Mar et al., 2015). However, in this model, the pressure pulse was applied to a focal region on the spine using a “blast gun,” aligning more closely with a contusive injury, the subjects were exposed to more than one pressure wave, representing a repetitive injury, and the durations were not representative of a typical free-field blast wave (between 1 to 10 ms) (Needham et al., 2015). Another group investigated a closed-body bSCI model up to 14 days after injury in rabbits, where necrosis and apoptosis of motor neurons were found to be greatest at 7 days post-injury. Additionally, motor function was significantly altered up to 14 days post-injury (Wang et al., 2010). These findings aid in understanding injury progression following blast exposure; however, the model demonstrated a close-range, focal blast with an extremely high average overpressure magnitude of over 7,000 psi, indicating this is a very severe injury model. Mild to moderate injuries were qualitatively investigated in a model for blast-induced neurotrauma in rats where evidence of axonal injury and membrane disruption were detected in the white matter tracts in the brainstem and cervical spinal cord at 6 and 24 h for a single overpressure of 22 psi (Kallakuri et al., 2015). These three studies provided evidence that an isolated blast wave can lead to axonal damage and detectable motor deficits. However, development of a more translational model of bSCI is needed.

In this preliminary work, we developed a translational closed-body model of blast-induced spinal cord injury, characterized the repeatability, and validated outcomes at 72 h following a single blast exposure through histological and behavioral measures where we were the first to investigate both glial and neuronal pathology in the spinal cord following blast exposure.

## 2. Materials and methods

### 2.1. Animal preparation

All animal protocols and procedures conducted herein were approved by the Virginia Tech Institutional Animal Care and Use Committee. Thirteen adult male Sprague Dawley rats (250–300 g; Envigo, Dublin, VA, United States) were housed under controlled conditions with a 12 h light:dark cycle where food and water were administered *ad libitum*. Based on a previous study, a minimum of six animals per group was used (Kallakuri et al., 2015). Animals acclimated for 1 week prior to experimental procedures. Subjects were 10 weeks old at the time of injury weighing an average of 262.0 ± 9.5 g.

On the day of the injury procedures, all animals were transported to the testing facility. Subjects were briefly anesthetized with 4% isoflurane through a surgical vaporizer connected to an induction chamber for procedural sedation. Weights were recorded, then a ketamine (80 mg/kg)/xylazine (10 mg/kg) solution was administered via intraperitoneal injection such that the animal remained anesthetized throughout the blasting procedure. Depth of anesthesia was ensured by checking the toe pinch reflex prior to blast testing. As thoracic and lumbar regions are most commonly injured following blast (Schoenfeld et al., 2013; Bernstock et al., 2015), the thoracolumbar region was chosen as our region of interest (ROI). The backs of the animals were shaved along the length of the spine and the vertebrae in the ROI were palpated and labeled with a permanent marker. Marking the skin provided us with an approximate region to look for when placing the animals in the bSCI fixture and the final position was determined and confirmed by palpating the vertebrae in the fixture opening.

### 2.2. Blast-induced SCI procedures

**Figure 1:**
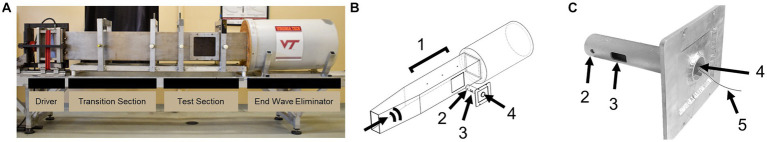
**(A)** Virginia Tech Advanced Blast Simulator (ABS) with labeled sections. **(B)** Annotated ABS with bSCI fixture denoting the direction of the blast wave. **(C)** bSCI fixture adapted to screw into the test section of the ABS. (1) PCB sensors measuring static overpressure in the wall of the ABS, (2) PCB sensor measuring the reflected pressure on the bSCI fixture, (3) The exposed spinal cord ROI, (4) Opening where the animals are inserted head-first on their left side in the bSCI fixture so that the thoracolumbar region fits in the ROI, and (5) Protected wire from the PCB sensor exiting the fixture.

The Virginia Tech Advanced Blast Simulator (ABS) was adapted with a custom bSCI fixture (Figure 1). Numerous studies have previously characterized the Virginia Tech ABS to investigate primary blast-induced brain and lung injury (Cho et al., 2013; Hubbard et al., 2014, 2015, 2017, 2018; Bailey et al., 2015; Sajja et al., 2015; Dickerson et al., 2020, 2021). The methods described here were adapted from previous methods by developing a bSCI fixture and aimed to focus the injuries to the spinal cord while minimizing surrounding organ damage, particularly to the lungs. In humans, primary blast lung injuries occur between 30 to 80 psi (Kang et al., 2012), however, lethality risk due to lung hemorrhaging was found to increase significantly in rats at overpressure magnitudes above 22 psi (Hubbard et al., 2014). The custom-built aluminum bSCI fixture (Figure 1C) was composed of two main elements. A ¼″ thick cylindrical tube was closed at one end and contained a 2″ x 1″ opening exposing the ROI to the blast wave. The cylindrical tube was welded to a rectangular mount that screws into the wall of the test section to secure the fixture in place and suspend the cylinder within the ABS.

Blast animals (*n* = 7) were then placed in the bSCI fixture by sliding the animal head first through the circular opening on the square mount until the shaved section of the spine was exposed. The spinal ROI was palpated once in the fixture to confirm positioning and mitigate rotation of the thoracic spine. The fixture was then secured into the test section and the animals were exposed to a single overpressure. Sham animals were placed adjacent to the test section of the ABS (*n* = 6). Vinyl membranes were passively ruptured following the pressurization of helium gas in the driver compartment. The blast wave traveled along the transition section, over the test section, and was dissipated in the end wave eliminator. Overpressures were collected at the three pressure sensors in the wall of the ABS next to the test section (PCB Piezotronics Inc., Model 102B16, Depew, NY, United States). Adding the fixture introduced reflections, which subsequently amplified the magnitudes measured by the wall sensors. For a more accurate measure of the reflected pressure experienced at the injury site, the fixture was instrumented with a piezoelectric pressure sensor (PCB Piezotronics Inc., Model 102B15, Depew, NY, United States) mounted adjacent to the exposed ROI. Immediately following the blast, Revertidine (atipamezole hydrochloride, 1 mg/kg SC) was administered to reverse the sedative effects of xylazine. The animals were monitored on a heating pad until awake and placed back in their cages.

### 2.3. Characterization of SCI model

Blast dynamics between subjects were analyzed to confirm the repeatability of the injury model using the pressure sensors mounted to the bSCI fixture and the wall of the ABS. Data was collected at 500 kHz on a Dash 8HF-HS system (Astro-Med, Inc., West Warwick, RI, United States) with a 4-channel signal conditioner (PCB Piezotronics Inc., Model 482C05, Depew, NY, United States) and analyzed in MATLAB (R2018b, The MathWorks Inc., Natick, MA, United States). Curves offset from zero were corrected and interpolation was performed in the case of data clipping. Data from one pressure trace was removed from the analysis due to severe clipping, likely from a pinched cable. To preserve wave characteristics, the pressure pulses were not filtered. The reflected pressure at the fixture ( $P_{ref}$ ) is related to the static overpressure in the wall of the ABS ( ΔP ) (Equation 1) where γ is the specific heat ratio of gas (for air γ =1.4) and $P_{0}$ is the atmospheric pressure ( $P_{0}$ =14.7 psi) (Needham et al., 2015). The percent difference between the measured and calculated reflected pressures was determined. The overpressure and reflected pressure magnitude, rise time, and duration of the positive phase were also calculated and compared.

$P_{ref}=2\Delta P+\left(\gamma +1\right)\left(\frac{5}{2}\times \frac{\Delta P^{2}}{7P_{0}+\Delta P}\right)$ **(1)**

### 2.4. Behavioral assessments

Subject behavior was monitored daily according to the Tarlov Motor Scoring system, ranging from 0 to 5, to assess the degree of functional neurologic impairment. A score of 0 indicates full paralysis, while a score of 5 indicates normal locomotion (Gale et al., 1985). Open Field Testing (OFT) was performed at 72 h following injury to further evaluate locomotor function and anxiety. As previously described by Dickerson et al. (2020), each subject was placed in an empty arena (80 cm^2^) and was allowed to explore for a total of 5 min. EthoVision XT (Noldus Information Technology, Leesburg, VA, United States) collected three-point tracking of the nose, center of the body, and base of the tail at 30 frames per second. The fraction of time spent in the center of the arena, maximum velocity, and total distance traveled were compared in the blast versus sham groups as indicators of anxiety-like behavior and locomotor deficits.

### 2.5. Immunohistochemistry

At 72 h post-injury, subjects were anesthetized with 4% isoflurane administered via induction chamber and depth of anesthesia was assessed by toe pinch reflex prior to euthanasia procedures. Subjects were euthanized via exsanguination followed by transcardial perfusion first with 0.9% saline then 4% paraformaldehyde. The lungs, spleen, kidneys, liver, and brain were extracted and inspected for visible signs of bruising or hemorrhaging. The spinal column was removed and post-fixed in 4% paraformaldehyde for 3 days. The spinal cord was then extracted from the spinal column and cryoprotected in a solution of 30% sucrose. Once fully submerged in the sucrose solution (~72 h), spinal cords were trimmed longitudinally into a 3 cm ROI. The ROI was embedded in Tissue-Tek optimal cutting temperature (OCT) compound, and frozen at -80°C. Positioning in the OCT block had to be very meticulous to consistently denote dorsal, ventral, rostral, and caudal directions. Once frozen, the spinal cords were cryosectioned along the sagittal plane at 20 μm thickness. Sections were stored at 4°C in 1X phosphate buffered saline (PBS) with 0.05% sodium azide solution until staining procedures.

Pathological outcomes were investigated in this bSCI model through histological staining of glial fibrillary acidic protein (GFAP), ionized calcium-binding adapter molecule 1 (Iba1), and S100 calcium-binding protein *β* (S100β) to assess glial reactivity and neuroinflammatory responses as well as neurofilament light chain (NF-L) and beta-amyloid precursor protein (β-APP) to assess traumatic axonal injury. Staining was performed on duplicate sections from the mid-sagittal plane of the spine (thickest). Details regarding staining procedures are provided in Table 1. Briefly, tissue sections were washed three times with PBS containing 0.3% Triton-X (PBX) for 3 min each and incubated in a blocking buffer. Sections were then incubated in the primary antibody of interest, which was diluted in blocking buffer overnight at 4°C. The following day, sections were rinsed three times with PBX for 3 min each and incubated in a secondary antibody diluted in blocking buffer (Life Technologies™, Grand Island, NY). After three washes in PBS, sections were incubated in a solution of 6-diamidino-2-phenylindole (DAPI; Sigma-Aldrich, St. Louis, MO, United States) diluted in PBS at a ratio of 1:10,000. After a final three washes in PBS, sections were mounted on slides, air-dried, and cover slipped with SlowFade™ Diamond Antifade Mountant (Invitrogen, Carlsbad, CA, United States). In addition to the steps previously mentioned, antigen retrieval was performed for the β-APP stain by first incubating sections in 0.3% H_2_O_2_ before the first PBX wash.

**Table 1:** Immunohistochemistry staining protocols.

Primary antibody (dilution)	Manufacturer	Catalog #	Blocking buffer (incubation time)	Secondary antibody (dilution; incubation time)
GFAP (1:500)	ThermoFisher	130300	10% Normal Goat Serum, 0.3% Triton-X (1 h)	Alexa Fluor™ 488 Goat Anti-Mouse (1:250; 1.5 h)
Iba1 (1:300)	Biocare Medical	Cp290a	10% Normal Goat Serum, 0.3% Triton-X (1 h)	Alexa Fluor™ 546 Goat Anti-Rabbit (1:500; 1.5 h)
NF-L (1:500)	Millipore	AB9568	10% Normal Goat Serum, 0.3% Triton-X (1 h)	Alexa Fluor™ 555 Goat Anti-Rabbit (1:250; 1.5 h)
S100β (1:1000)	Millipore	S2532	10% Normal Donkey Serum, 0.3% Triton-X (1 h)	Alexa Fluor™ 594 Goat Anti-Mouse (1:500; 3 h)
β-APP (1:250)	Invitrogen	51–2700	2% Normal Goat Serum, 0.3% Triton-X (2 h)	Alexa Fluor™ 555 Goat Anti-Rabbit (1:500; 1 h)

### 2.6. Imaging procedures

Fluorescent microscopy was performed on a Zeiss fluorescence microscope at 20X magnification, and images were captured with AxioCam ICc1 camera (Zeiss, Jena, Germany). A total of six representative images were taken between the duplicate tissue sections from each subject from rostral to caudal along the white matter of the spinal cord. The area fraction of positive signal, cell count, and fluorescent intensity were quantified using ImageJ software (National Institute of Health, Bethesda, MD, United States) (Schneider et al., 2012).

### 2.7. Statistical analysis

Behavior and immunohistochemistry statistical analyses were performed comparing sham and blast treatment groups using unpaired two-sample *t*-tests in GraphPad Prism 9 (GraphPad Software, San Diego, CA, United States) where *p* < 0.05 was considered significant.

## 3. Results

### 3.1. Blast characterization

**Figure 2:**
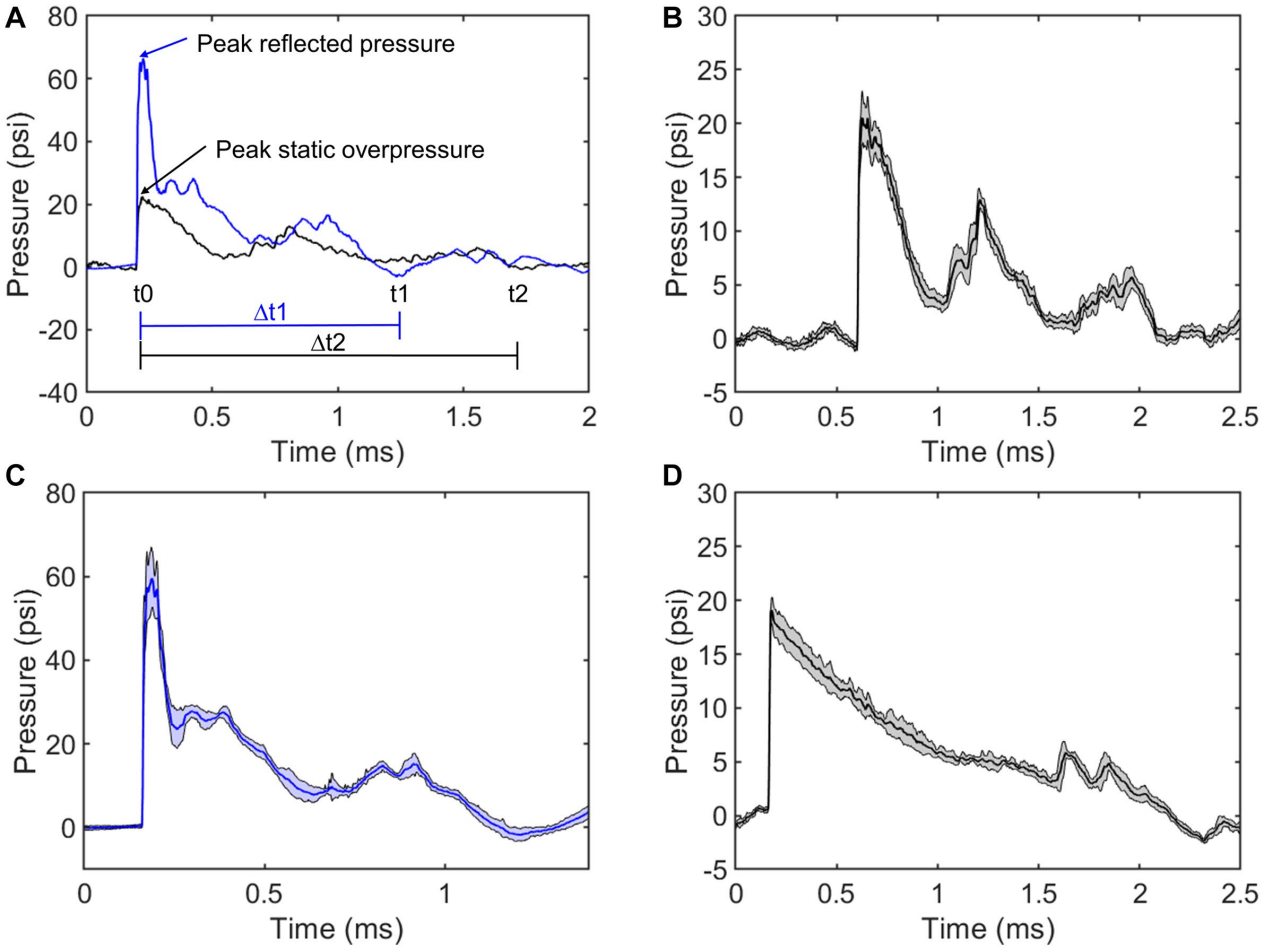
Comparing repeatability of static overpressures in the ABS (black) and reflected pressures on the bSCI fixture (blue). **(A)** Representative static overpressure and associated reflected pressure. Annotations include peak pressures and positive durations (∆t). **(B)** Average static overpressure ± standard deviation adjacent to the bSCI fixture. **(C)** Average reflected pressure ± standard deviation in the bSCI fixture. **(D)** Average static overpressure ± standard deviation upstream from the test section in the ABS.

The average pressure traces were provided for visual inspection (Figure 2). Figure 2A shows the annotated positive durations and peak pressures. The average static overpressure and reflected pressure traces directly adjacent to the subject are shown in Figures 2B,C, and the average static overpressure trace upstream of the bSCI fixture is provided in Figure 2D. To best replicate a free-field blast scenario, the ideal pressure pulses along the ABS should maintain a Friedlander waveform with a static overpressure positive phase duration of approximately 2 ms. The static overpressure upstream of the fixture follows a consistent Friedlander waveform with an average peak overpressure of approximately 20 psi with a 2 ms duration. However, as the wave interacted with the fixture, the overpressure adjacent to the fixture was noisier than the upstream overpressure, and reflections from the fixture amplified the magnitude directly adjacent to the exposed spinal cord ROI.

To further characterize the repeatability, the average positive duration, rise time, peak magnitude, and positive impulse were calculated (Table 2). The positive impulse was calculated as the area under the curve throughout the positive duration and the rise times were calculated as the time from t_0_ to respective peak pressures. As previously mentioned, the wave characteristics between the upstream static overpressure and that adjacent to the fixture were distinctly different where the adjacent overpressure positive duration decreased, the rise time and its standard deviation increased, and peak magnitude and standard deviation increased, which are all counterintuitive and are driven by reflections from the fixture. The measure of reflected pressure on the bSCI fixture was a more robust way to measure pressures at the site of injury. This was confirmed by comparing the percent difference between the calculated reflected pressures (using Equation 1) based on the static overpressure adjacent to the fixture and the measured reflected pressures on the fixture. The percent difference ranged from 0.7 to 14.3%. While these percentages are low, they are also highly variable and indicate that the reflections from the fixture may introduce extra variability in the measured static overpressure. Nevertheless, the measured static overpressure and reflected pressures displayed minimal variability, consistent with the unobstructed sensors upstream.

**Table 2:** Blast wave characteristics comparing the static overpressure upstream of the bSCI fixture (Upstream), static overpressure adjacent to the bSCI fixture (Adjacent), and the reflected pressures within the bSCI fixture (Fixture).

Sensor locations	Average positive duration (ms)	Average rise time (ms)	Average peak magnitude (psi)	Average positive impulse (psi^*^ms)
Upstream	1.99 ± 0.03	0.019 ± 0.002	19.18 ± 1.24	14.03 ± 0.66
Adjacent	1.56 ± 0.09	0.030 ± 0.011	20.88 ± 2.10	9.84 ± 0.39
Fixture	1.21 ± 0.54	0.027 ± 0.002	59.93 ± 7.12	16.70 ± 1.24

### 3.2. Behavioral validation

**Figure 3:**
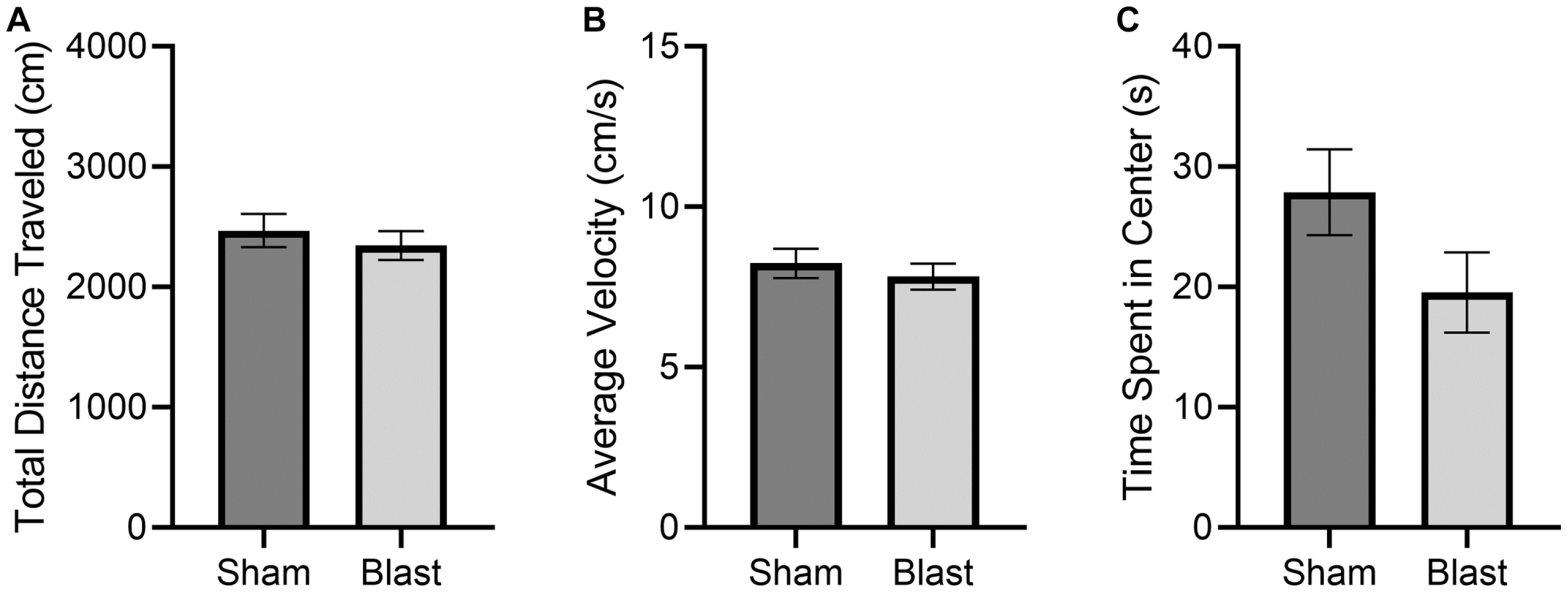
OFT results at 72 h comparing the mean ± SEM of the sham and blast treatment groups for the **(A)** total distance traveled in the arena (cm), **(B)** average velocity (cm/s), and **(C)** cumulative time spent in the center of the arena (s) (*p* = 0.1163). No significant differences were found.

Normal locomotion was observed according to Tarlov’s Scale for all subjects. No significant differences were observed in the OFT results between the sham and blast groups (Figure 3). There was less activity in the center of the OFT arena, although this was not significant. Less activity in the center is an indication of increased anxiety and has been previously detected as early as 48 h following bTBI (Hubbard et al., 2018).

### 3.3. Histological validation

**Figure 4:**
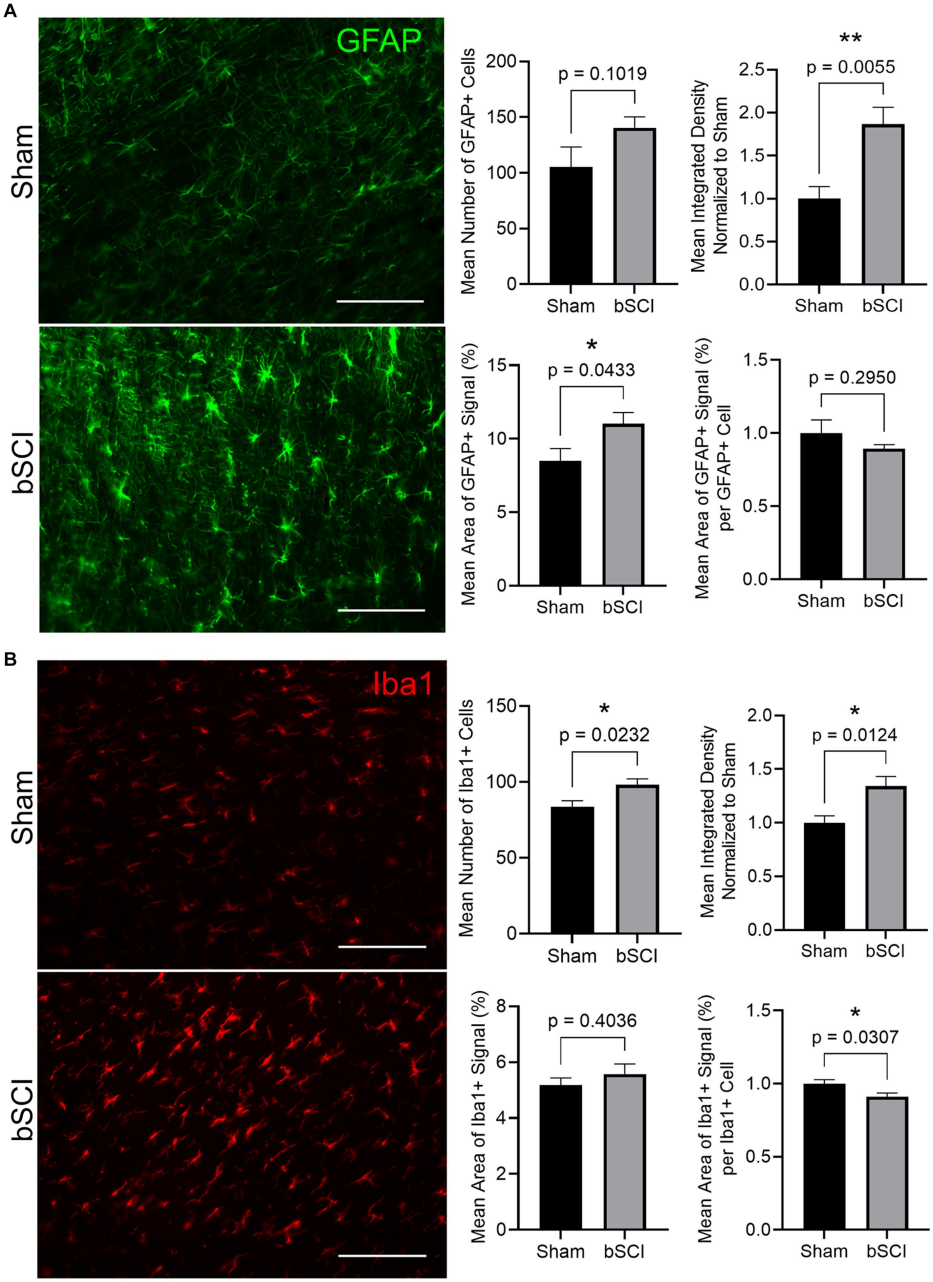
Representative images comparing sham and blast treatment groups at 72 h post-injury provided for markers of glial reactivity (scale bar = 150 μm). Bar graphs show the mean ± SEM. **(A)** GFAP area fraction (^*^p < 0.05) and integrated density (^**^p < 0.01) significantly increased following blast while there was no significant difference in number of GFAP-positive cells or area of signal per cell. These changes are representative of astrocyte reactivity. **(B)** Integrated density and number of Iba1-positive cells significantly increased while the area of positive signal per cell significantly decreased (^*^p < 0.05). These changes are representative of microglia activation and migration.

Markers of glial reactivity and neuroinflammation were significantly altered following bSCI. Evidence of astrocyte reactivity was indicated by the significant increase in the mean GFAP integrated density as well as the mean area of positive GFAP signal. Additionally, there was a trending increase in the number of GFAP-positive cells (Figure 4A). Expression of Iba1 was quantified in the spinal cord as a measure of microglial reactivity. A significant increase in Iba1 integrated density combined with a significant decrease in area of positive Iba1 signal per microglial cell was quantified (Figure 4B), which is indicative of microglial reactivity. Additionally, the mean number of Iba1-positive cells increased, which has been linked to cell migration and neuroinflammation at the injury site.

**Figure 5:**
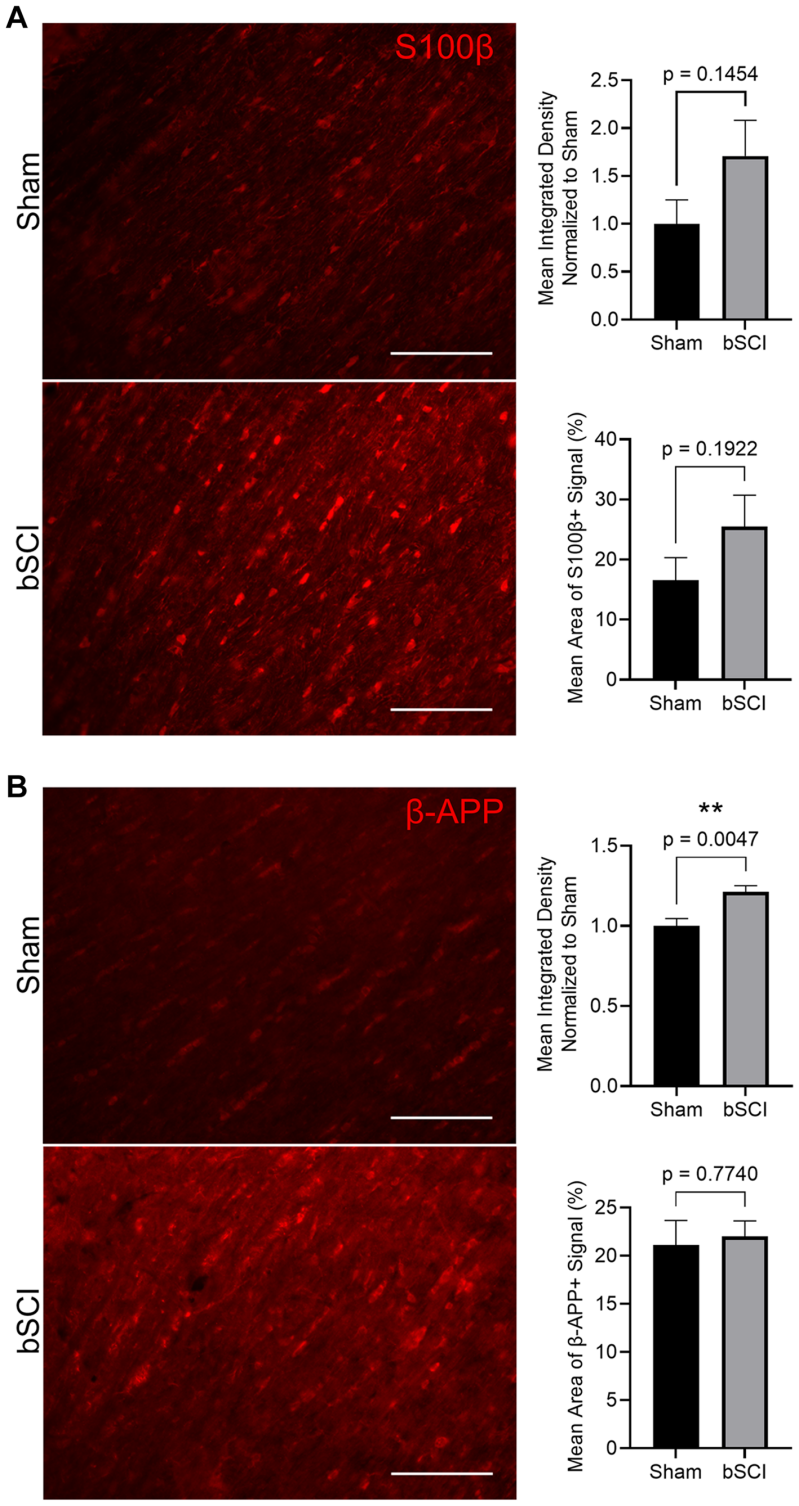
Representative images of comparing sham and blast treatment groups at 72 h post-injury provided for injury biomarkers (scale bar = 150 μm). Bar graphs show the mean ± SEM. **(A)** Average increase in area fraction and integrated density of S100β, however, changes were not significant. **(B)** Significantly increased β-APP integrated density (^**^p < 0.01) with no change in the average area of positive signal. This indicates a more concentrated change in expression within the axon.

The change in area fraction and integrated density of S100β and β-APP were quantified in Figure 5. Elevated expression and area of positive signal have been linked to injury severity, making these promising biomarkers. There was an increase in the mean positive S100β area and fluorescence intensity, however, these values were not significant (Figure 5A). Alternatively, β-APP expression was significantly increased compared to sham (Figure 5B). This increase in β-APP expression without a change in positive signal area may indicate that axonal swelling is present without secretion of β-APP from the axon. Further investigation of traumatic axonal injury, which is characterized by axonal swelling and impaired axonal transport, was investigated through quantification of NF-L. At 72 h post-blast, no significant changes were detected in the NF-L area fraction or integrated density, and thus cytoskeletal disruption was not evident at this time point (Supplementary Figure S1).

## 4. Discussion

This work developed a novel model to investigate pathological outcomes in the spinal cord following exposure to a blast wave, which was motivated by the need for improved clinical outcomes following bSCI. The mechanism of this injury results from direct exposure to a blast wave and may be followed by blunt force trauma and/or chemical effects from an explosion, leading to complex pathologies and poor clinical outcomes. It is common practice for preclinical models of SCI to perform a laminectomy as part of the injury model, which can potentially introduce confounding pathophysiological effects that are inconsistent with the types of injuries seen on the battlefield. Further, the laminectomy protocols require extensive time for the animals to be under anesthesia, which is also a concern. Therefore, we aimed to utilize a closed-body approach without a laminectomy to study bSCI that reduced the length of time under anesthesia (<10 min) and eliminated the need for ventilation of animals. Two previous studies reported investigating bSCI axonal pathology as well as behavioral outcomes following directed blasts to a focal region of the spinal cord (Wang et al., 2010; del Mar et al., 2015). However, the biomechanical testing involved multiple high magnitude, long-duration, focused pressures, which are not directly translational to explosive mechanisms experienced on the battlefield (del Mar et al., 2015). Additionally, injuries from close-range exposure to over 7,000 psi are more likely to cause severe trauma to the body, resulting in death (Wang et al., 2010). The closed-body model in this study was developed to overcome these disadvantages. We replicated free-field blast dynamics and quantified the repeatability through a detailed blast analysis using the Virginia Tech ABS, which created upstream pressure pulses following a Friedlander waveform with an average positive duration of 1.56 ± 0.09 ms and an overpressure magnitude of 20.88 ± 2.10 psi adjacent to the ROI across all blasts. This target pressure was determined based on a previous study that found qualitative evidence of axonal damage in the brainstem and cervical spine following exposure to 22 psi (Kallakuri et al., 2015) and led to the hypothesis that the thoracolumbar region of the spinal cord is susceptible to unique, diffuse pathology following a single blast exposure, similar to that seen in the brain.

Blast-induced traumatic brain injuries (bTBI) are widely studied and known to have a unique pathological response following exposure to a blast wave (Wolf et al., 2009; Hicks et al., 2010; Sajja et al., 2012, 2014, 2015; Cho et al., 2013; Bailey et al., 2015; Cernak, 2015; Hubbard et al., 2017; Dickerson et al., 2020). In particular, chronic bTBI studies demonstrated that blast exposure leads to a long-term inflammatory response and associated anxiety-like behavior in rats up to 1 year post bTBI (Arun et al., 2020; Dickerson et al., 2021). While the exact mechanism of injury remains unclear, it is certain that the skull does not prevent the brain from experiencing a rapid increase in pressure, eliciting diffuse stresses and strains that cause damage to the brain tissue (Fievisohn et al., 2018). Therefore, even protected by the spinal column, we anticipated that the blast wave caused a similar pathological response along the spinal cord.

Preliminary bSCI studies each showed axonal swelling within the first 24 h following the blast; however, the most striking motor deficits occurred between 1- and 6-days post-injury and differences were detected up to 3 weeks (Wang et al., 2010; del Mar et al., 2015; Kallakuri et al., 2015). We performed a separate acute study (*n* = 5 sham, *n* = 4 blast) to compare OFT results at 24 and 72 h. This showed a significantly decreased (*p* = 0.0189) frequency of movements to the wall of the OFT arena in the 72 h blast group compared to 24 h (Supplementary Figure S2). Thus, 72 h was selected as the time point of interest for this study. While no significant motor differences were detected between the blast and sham groups at 72 h, it is unclear whether behavioral responses would be altered at later time points or if the injury severity was just not significant enough to elicit any noticeable changes in the locomotion. Investigating behavior at chronic time points post-injury may present more obvious motor dysfunction as the secondary injury cascades continue unabated. Alternative motor tests such as rotarod, beam walk, or grid walk, as well as sensory behavior experiments should be considered in future studies to capture a wider range of behavioral deficits commonly associated with spinal cord injuries.

Axonal swelling, as indicated by elevated levels of β-APP, was present in the thoracolumbar region of the spinal cord at 72 h following bSCI. However, there was no indication of cytoskeletal disruption. Previous studies found evidence of neurofilament accumulation in the cervical and thoracic spinal regions within the first 24 h following blast (del Mar et al., 2015; Kallakuri et al., 2015), indicating the window for maximal cytoskeletal disruption may be earlier than 72 h and recovery is ongoing. On the other hand, it is possible that elevated β-APP could be propagated by the glial response (Montagna et al., 2017). Colocalization between neuronal swelling and the microglial and astrocytic response should be further investigated to determine if elevated β-APP is linked to structural damage or the inflammatory response.

While previous bSCI studies focused on neuronal survival, this work was the first to investigate the combined glial and neuronal pathology in the spinal cord resulting from a single blast exposure. Similar to bTBI, our findings confirm that this pathology is glial driven with elevated expression of Iba1 and GFAP beyond 48 h post bSCI (VandeVord et al., 2012; Cho et al., 2013; Hubbard et al., 2017; Dickerson et al., 2020). However, we did not detect significant changes in S100β expression at 72 h where increased expression of S100β is known to stimulate the production of pro-inflammatory cytokines and contribute to neuroinflammatory cascades. Studies have indicated that S100β is a less sensitive measure of gliosis compared to GFAP expression (Kaur and Conti, 2021) and has a delayed increase in expression following reactivity of GFAP. In study by de Menezes et al., increased GFAP expression was greatest at 48 h post-injury and S100β expression reached its peak at 1 week (de Menezes et al., 2018). Additionally, neuroinflammation of the spinal cord was characterized by increased expression of GFAP, Iba1, and S100β at 2 weeks following lumbar puncture where it was found that changes in expression were spatially dependent and varied from dorsal to ventral (Kaur and Conti, 2021). Future work should determine the spatial and temporal dependence of GFAP, Iba1, and S100β expression after bSCI to better define this neuroinflammatory cascade.

A recent study showed that astrocytes proliferate following a repetitive TBI and that the process was promoted by the migration of monocytes to the location of the focal injury (Lange Canhos et al., 2021). Other studies suggest that astrocytes migrate following trauma to the injury site in both a TBI (Lagos-Cabré et al., 2020) and an impact SCI model (Renault-Mihara et al., 2011). While our model produces a diffuse mild SCI without impact, as opposed to a severe focal injury, our data demonstrated an increased number of Iba1-expressing cells and increased Iba1 expression level. We further found that as the expression level of GFAP significantly elevated and, the mean number of GFAP-positive cells increased (although not significant). Taken together, the data from our bSCI model suggests that astrocytes may be proliferating at this early time point in response to inflammation, however, we cannot exclude the possibility that they are migrating. Our data also demonstrated that microglia were migrating and undergoing morphological changes in the ROI. Future directions will include a detailed longitudinal investigation of migration and proliferation using this bSCI model.

Increased glial reactivity is also known to be associated with oxidative stress. Reactive oxygen species (ROS) become activated in macrophages and glial cells following injury where oxidative stress results from elevated antioxidant defenses. Further quantification of the association between ROS production in microglia and astrocytes as well as any changes in morphology could aid in understanding the interplay between neuroinflammation and oxidative stress following bSCI.

When considering a holistic approach to the bSCI, it is likely that the propagation of the blast wave also leaves other critical units of the nervous system, such as the spinal nerves, susceptible to damage. When spinal root avulsions occur, they can lead to loss of sensory and autonomic function or paralysis, but most commonly leads to neuropathic pain (Aldskogius and Kozlova, 2021). One study investigated the relationship between the severity of nerve root deformation and the extent of microglial and astrocyte response. It was found that the more severe the nerve root deformation, the greater the microglial activation. Astrocytic response was not found to be dependent on the severity (Winkelstein and DeLeo, 2002). Since activation and proliferation of microglia following damage to the nerve root is a common response, it is unclear how much of the neuroinflammatory response could be triggered by damage to the nerve roots and this should be further investigated (Xue et al., 2014).

We established a repeatable closed-body preclinical model of bSCI which is the first to provide evidence of a glial-driven response in the spinal cord following blast injury. We have shown that this injury model did not limit overt locomotion or result in significant acute neuronal damage, however, axonal swelling and glial reactivity at 72 h post-injury indicated mild inflammatory pathology. Future directions that could be considered to further characterize this novel injury model include examining chronic time points and the use of a large animal model to improve translational aspects of the study. Longitudinal effects should be quantified through histology and behavioral assessments to better define this pathophysiological response and sex-specific differences should be taken into account. These findings could then be validated in larger animal models (such as porcine) to demonstrate translational and clinical relevance between models. Integration of blood serum and cerebrospinal fluid collection across longitudinal time points may also prove beneficial for injury characterization. Further investigation of the unique pathological outcomes resulting from bSCI will be performed to examine the longitudinal effects of blast exposure on neuroinflammation and associated behavior where this model can be applied to test treatment approaches for systemic neuroinflammation in the spinal cord.

## Data availability statement

The raw data supporting the conclusions of this article will be made available by the authors, without undue reservation.

## Ethics statement

The animal study was reviewed and approved by the Virginia Tech Institutional Animal Care and Use Committee.

## Author contributions

CN: conceptualization, methodology, software, validation, formal analysis, and writing – original draft. JW: methodology, validation, and writing – reviewing and editing. SM: conceptualization, methodology, formal analysis, and writing – reviewing and editing. IM and LM: investigation and writing – reviewing and editing. AB and PV: funding acquisition, supervision, conceptualization, methodology, and writing – reviewing and editing. All authors contributed to the article and approved the submitted version.

## Funding

This study was supported by the Research Eureka Accelerator Program (REAP) Award #1038509 issued from the Edward Via College of Osteopathic Medicine (VCOM) and Virginia Tech Institute for Critical Technology and Applied Science (ICTAS) partnership.

## Conflict of interest

The authors declare that the research was conducted in the absence of any commercial or financial relationships that could be construed as a potential conflict of interest.

## Publisher’s note

All claims expressed in this article are solely those of the authors and do not necessarily represent those of their affiliated organizations, or those of the publisher, the editors and the reviewers. Any product that may be evaluated in this article, or claim that may be made by its manufacturer, is not guaranteed or endorsed by the publisher.
