# Supplementary material for: A closed-body preclinical model to investigate blast-induced spinal cord injury
Source: Front Mol Neurosci. 2023 Jun 13;16:1199732. doi: 10.3389/fnmol.2023.1199732 (PMC10293620; doi:10.3389/fnmol.2023.1199732)
Supplement: Supplementary file 1 [file Data_Sheet_1.DOCX]

Supplementary Material

A Closed-Body Preclinical Model to Investigate Blast-Induced Spinal Cord Injury

Carly Norris, Justin Weatherbee, Susan Murphy, Izabele Marquetti, Lana Maniakhina, Alan Boruch, and Pamela VandeVord^*^

***Correspondence:** Dr. Pamela VandeVord: pvord@vt.edu

# Supplementary Figures and Tables

## Supplementary Figures


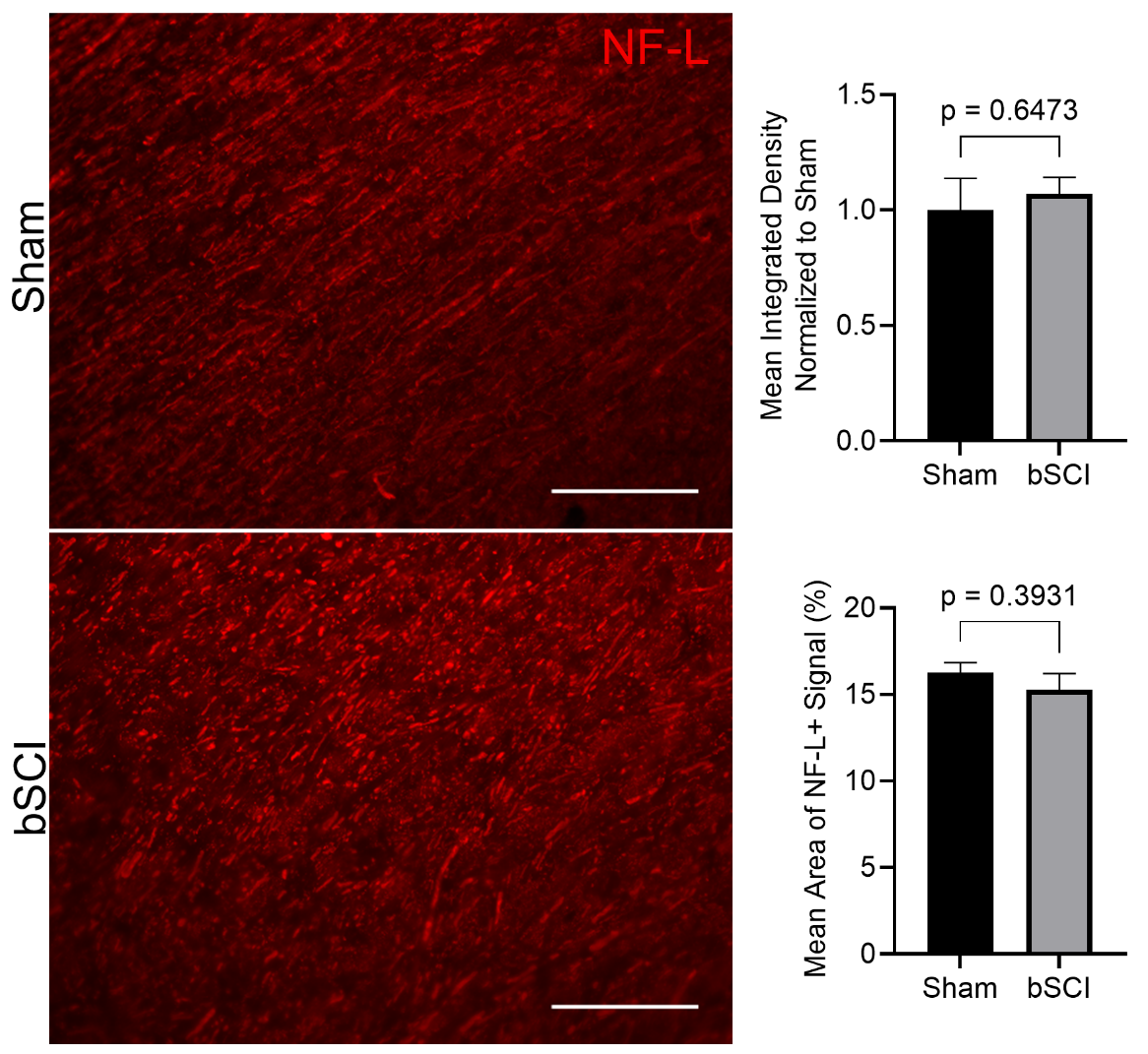


**Supplementary Figure 1.** Representative images comparing sham and blast treatment groups at 72 hours post-injury provided for NF-L, a marker of traumatic axonal injury (scale bar = 150 μm). Bar graphs show the mean ± SEM. NF-L area fraction and integrated density did not significantly change at 72 hours following the blast. Therefore, axonal damage leading to accumulation, elevated expression, or secretion was not detected at this time point.

**
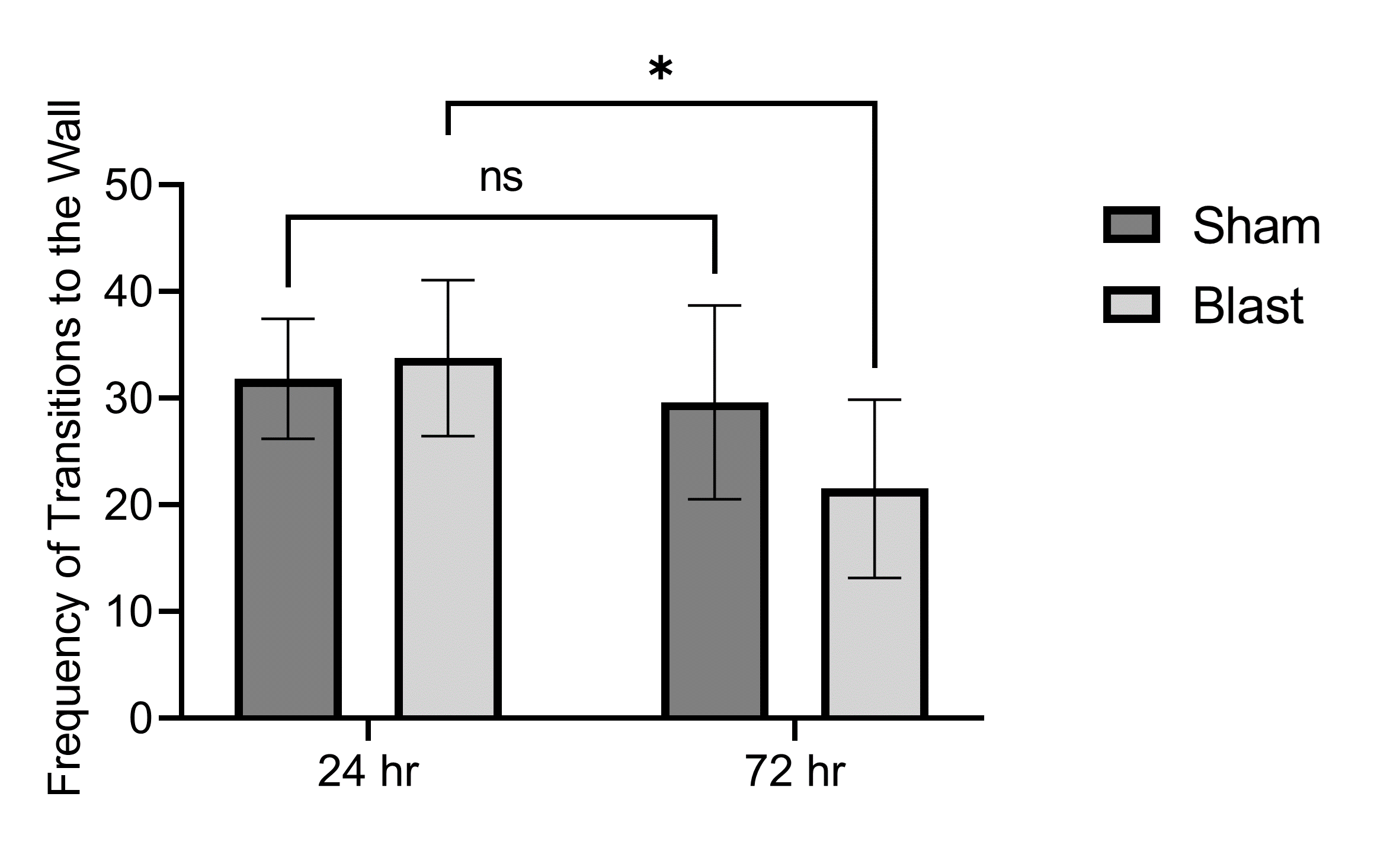
**

**Supplementary Figure 2.** OFT preliminary results comparing the mean ± SEM of the wall frequency between sham (n=5) and blast (n=4) treatment groups following bSCI exposure where there was a significant decrease in frequency of transitions to the wall of the arena in the 72 hour blast group compared to the 24 hour blast group (p=0.0189).
